# Design of a Diagnostic System for Patient Recovery Based on Deep Learning Image Processing: For the Prevention of Bedsores and Leg Rehabilitation

**DOI:** 10.3390/diagnostics12020273

**Published:** 2022-01-21

**Authors:** Donggyu Choi, Jongwook Jang

**Affiliations:** Department of Computer Engineering, Dong-Eui University, Busan 47340, Korea; dgchoi@office.deu.ac.kr

**Keywords:** deep learning, artificial intelligence, diagnostic program, rehabilitation, human pose estimation

## Abstract

Worldwide COVID-19 infections have caused various problems throughout different countries. In the case of Korea, problems related to the demand for medical care concerning wards and doctors are serious, which were already slowly worsening problems in Korea before the COVID-19 pandemic. In this paper, we propose the direction of developing a system by combining artificial intelligence technology with limited areas that do not require high expertise in the rehabilitation medical field that should be improved in Korea through the prevention of bedsores and leg rehabilitation methods. Regarding the introduction of artificial intelligence technology, medical and related laws and regulations were quite limited, so the actual needs of domestic rehabilitation doctors and advice on the hospital environment were obtained. Satisfaction with the test content was high, the degree of provision of important medical data was 95%, and the angular error was within 5 degrees and suitable for recovery confirmation.

## 1. Introduction

Recently, COVID-19 has caused various problems in countries throughout the world and Korea is no exception, it is currently going through an extremely challenging medical period. Medical care, along with the economy, is facing very serious problems. In addition to the aging society, the number of economically active people is becoming scarce, and the welfare and medical fields to support it are becoming increasingly vulnerable [1,2,3,4]. In particular, compared to the increasing number of patients, medical personnel were insufficient, and in the existing case, it was difficult to accommodate all patients, so they were often unable to receive the necessary emergency measures. There is a demand for being face-to-face with patients, and the medical field that requires the most time and uses alternative personnel for treatment activities in the medical field is rehabilitation.

Diagnosis and treatment related to rehabilitation requires practical manpower, and due to continuous manpower shortages, services for patients in need of further treatment are becoming insufficient. In fact, to illustrate the current situation as an example, the number of patients due to the coronavirus is increasing exponentially and there is an increasing lack of beds manpower to care or assist them [5,6,7]. In order to address this, public officials or volunteers are mobilized to provide support for possible areas other than services provided by medical staff, and even if it is not an emergency, problems arise depending on the number of different medical personnel and the ever-decreasing medical personnel in each hospital [8,9].

The rehabilitation medical field includes all medical practices performed by mobilizing care or nursing personnel regarding general doctor examinations and treatments [10]. Even in this case, there is a need for 1:1 management of the patient, so even if it is performed by alternative personnel, a deficiency could continue to exist, for example, bedsores. Bedsores can become serious if they do not receive attention [11,12,13]. After surgery, patients with disabilities or elderly patients are likely to develop the lesions because it is difficult to control their bodies at will and most of them lie still for prolonged periods. If bedsores are not prevented, even if there is no immediate problem, the blood does not circulate smoothly, resulting in rotting flesh and requiring another treatment for secondary infection or additional treatment.

The aforementioned problems and factors are only a few that exist. This is because the category of rehabilitation is wider and is generally not limited to exercise-related cases [14]. The entire process of curing the disease, that is, all tasks associated with it, such as relaxation in an uncomfortable state, are considered rehabilitation. In the case of patients who are difficult to move, a recognized way to strengthen the body for rapid recovery is by simply performing movements to the extent that they think they have moved their arms and legs. This simple movement is actually widely used as a strengthening method to improve the intensive pain of patients after surgery and increase the range of motion of the reduced joint, which includes checking how far the patient’s recovery has progressed through this process [15,16,17,18].

Recently, various integrated technologies have emerged based on the Fourth Industrial Revolution Technology are being utilized by the medical field to try to solve related problems by improving service quality. Although they are trying to assist some of the aforementioned medical practices, it is difficult to introduce medical laws and regulations because they can only be introduced with the support of experts. VUNO (Seoul, South Korea) is a company that is researching and developing related technologies using artificial intelligence in Korea [19].

**Figure 1:**
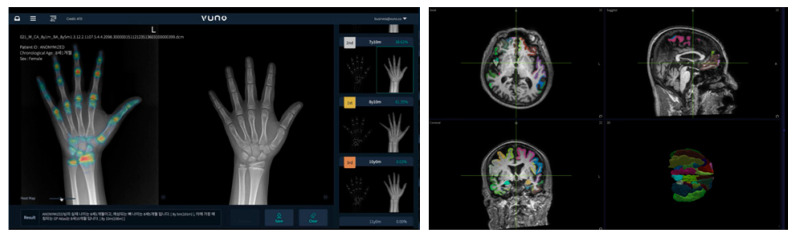
The image of VUNO’s artificial intelligence diagnosis program.

Figure 1 shows artificial intelligence diagnosis programs developed by VUNO. VUNO developed ‘BoneAge’, the first artificial intelligence medical device in Korea, and was registered as a medical device with permission from a related state agency in Korea in May 2018. ‘BoneAge’ is a ‘diagnosis assistance program’ that automatically analyzes X-ray images of hand and wrist bones and presents the most similar bone age up to the third place within seconds to help medical staff make a diagnosis [20]. In addition, a total of six programs have been developed, including ‘DeepBrain’ to check the degree of brain atrophy, ‘Chest X-ray’ to help diagnose chest X-rays, ‘Fundus AI’ to diagnose retinal diseases based on fundus images, ‘LungCT AI’ to detect lung nodules in real-time, and ‘DeepASR’ to recognize the voices of medical staff [19]. However, most of these programs are only used to assist medical diagnosis; they cannot be used to make a final diagnosis decision on their own because there is a problem of accuracy.

Table 1 compares two representative solutions used in Korea. Since the introduction of AI into the medical field is not yet legally easy, there is limited AI certified for use. In the case of VUNO, direct or indirect diagnostic solutions using medical images are generally provided. In the case of Watson from IBM, the patient’s symptoms are used as data to provide diagnostic results. However, Watson raised the issue of accuracy during use and is now suspended. VUNO is the first domestic deep learning diagnostic solution that is currently certified and in use. Overseas, development is being attempted to diagnose various lesions using deep learning, and artificial intelligence diagnostic research is underway to solve schizophrenia and epileptic seizures using brain waves and brain photos, starting with the currently problematic pulmonary X-ray of COVID-19 [21,22,23,24,25,26].

In order to solve some of the problems, such as the current shortage of medical personnel in Korea, this paper proposes a method of producing and utilizing system demonstrations using deep learning image processing technology to check the degree of diagnosis and the recovery of rehabilitation-related areas. Measurement solution content was produced and tested to solve the time-consuming part of performing patient management directly or using a protractor to identify joint-related lesions. Due to the law or the accuracy of artificial intelligent medical devices, advice was sought through actual hospitals on how to help medical staff using related technologies and areas not limited to medical law. The method involves flipping the body to prevent bedsores from occurring in patients who lie down after surgery and checking how well their arms and legs have been moved. The system proposed in the paper can be used even if it is not a hospital, depending on the installation method or location.

## 2. Related Works

In conducting the study, it was necessary to check the design method, and the application method according to the object identification method of deep learning was considered. Joint tracking and object detection methods were combined to check whether the motion was in progress, and angular calculations were performed based on the coordinates obtained through joint tracking.

### 2.1. Human Pose Estimation

Various technologies using computer vision are used, and what will be used in this study is human pose estimation. It is also called joint tracking, which means the technique of recognizing a person’s posture. It is a method of estimating the location of a person’s body joint in a photo or video. However, it is a difficult technique to identify joints only with images, and it is also tracked differently depending on the person doing the operating and the camera shooting direction. It is also a difficult technology field because the variables are so diverse.

**Figure 2:**
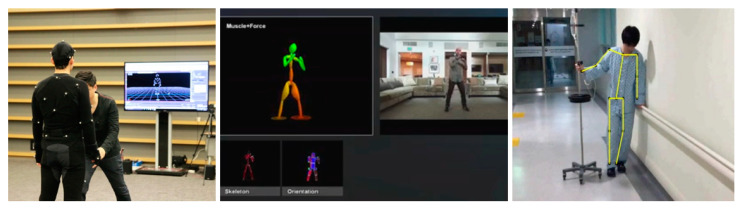
Various human pose estimation technology images.

Figure 2 shows various joint tracking methods. From the left, it shows the method of attaching a sensor to the body, the method through a 3D camera, and the result of using a general camera [27,28,29].

Table 2 shows the practicality of each joint tracking method. Although the sensor-attached measurement method is sophisticated, it is expensive in the initial stage and having to wear equipment for each measurement is an inconvenience. However, although the method of using a camera does not require any additional equipment, 3D cameras have the disadvantage of being more expensive than 2D cameras, and joint tracking performance is very limited compared to the sensor-attachment method. Furthermore, in the case of 2D cameras, additional technology or hardware is required because data is insufficient to recognize three-dimensional coordinates. Even if the joint is tracked, only the coordinates are displayed in the virtual space, and data such as the angle of the joint require additional computational work.

This study was conducted using deep learning. It is largely divided into two-dimensional and three-dimensional measurements and refers to a method of tracking data on a plane and a method of including depth data, such as using hardware. There are top-down and bottom-up methods for tracking. Top-down is a method of detecting a person in an image first and then estimating a person’s posture inside a bounding box. If a person is not recognized, the posture cannot be measured, and as the number of people increases, the number of calculations increases. The bottom-up method can cause problems in close joint matching by recognizing joint parts and connecting them to each other to estimate posture; however, there is no process for detecting humans, so it is suitable for real-time processing.

**Figure 3:**
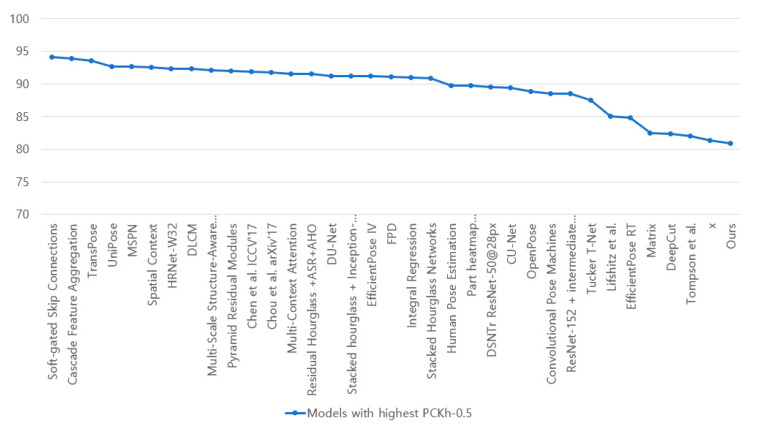
Accuracy by deep learning model using MPII human pose dataset [30].

Figure 3 shows the accuracy of the current pose estimation codes. Accuracy uses an indicator called “Percentage of Correct Keypoint” using the MPII Human pose Dataset and is judged as True Positive when the estimated coordinates and correct coordinates are less than any threshold at the joint point. It is the same indicator as the general AP indicator used in the COCO dataset. OpenPose does have high accuracy but adopts a bottom-up method that is effective for real-time utilization. It was confirmed that there was no problem with this accuracy in confirming the validity of the content by adding a joint angle measurement function.

Attempts to incorporate this human pose estimation technology into the medical field continues [31,32,33]. Usually, since it is a technology that mainly checks movement, research is mainly conducted to track joints that do not work properly and check movements such as joint movement range. Studies in slightly different directions were also conducted, and methods of identifying problems in the patient’s daily movements or using them for the mental treatment of patients were also conducted [34].

### 2.2. The Seriousness of Bedsores and the Need for Rehabilitation

Bedsores are caused by the weight of the body blocking the blood supply when sitting or lying in one position for too long if people cannot move well. In ordinary patients, it occurs when sitting in a bed or wheelchair for a long time. If it is detected in advance, before a pressure ulcer occurs, it can be cured by moving the body little by little; however, for some patients it may be difficult to move even a little. In this case, it gets worse and worse, forming pus in the area and even resulting in rotting.

Bedsores are not generally disease-inflicted, but problems that occur in life, often caused by patient management issues in hospitals, and they are common regardless of gender or age. Bedsores can be prevented with simple treatment and/or prescriptions, yet it is still a significant problem within the current medical community where the number of management personnel is gradually decreasing. In particular, the problem is getting more serious as the population of the elderly who are vulnerable to bedsores is increasing. Recently, to solve this problem in Korea, the world’s first monitoring system to prevent bedsores with wireless battery-free soft pressure sensors has been developed [35]. However, most patients are still directly identified and managed, and diagnosis is performed using a protractor, especially for identifying lesions related to joint measurement.

In addition, COVID-19 has increased the number of patients with respiratory organs, and since the respiratory tract is also a factor that puts pressure on the oral cavity, bedsores occur. Since bedsores are diseases caused by pressure, not only do they occur when the body is leaning against something, they also occur in various ways depending on the patient’s condition [36].

Most of the patients with problems often have difficulty controlling their bodies, which is caused by musculoskeletal disorders or surgery in related areas but weakening muscle strength is not unconditionally due to surgery and illness. Concerning the human musculoskeletal system, to ensure healthy functioning, the legs should be continuously used for basic movement; however, people are increasingly sitting down more often and for longer and, therefore, muscle strength of the legs is becoming weaker. Legs are an important part of the human anatomy for movement and a healthy and full life and are used with frequency, hence, they are exposed to problems. In addition, some researchers have studied these problems to prevent sarcopenia or hypertrophic cardiomyopathy in people with poor movement skills. They studied the correlation between long-term hospitalization in nursing homes and the drugs, senile syndrome, and behavior present in home care [37,38,39]. Another study was conducted to confirm the importance of leg muscle strength and to provide rehabilitation programs that can be performed at home for patients with weak leg muscle strength using an online system. Methods have also been studied using related robotics and IoT technologies, and various attempts are being made to solve some of the rehabilitation-related processes that do not have to be performed by medical personnel or in hospitals [40,41,42,43]. Patients rely on rehabilitation treatment to return to their daily lives, and rehabilitation through exercise is an essential factor in medical practice because it provides various solutions along with medication.

## 3. Design

The program was designed using OpenPose and AlphaPose, created using Python, and are used for joint tracking [29,44]. For fast processing speed, MobileNet was used as the backbone of the neural network [45]. The MPII Human Pose dataset was used as the dataset used to perform learning. The associated hyper parameters were performed equally without significant changes. In a recent method, depth data can also be expressed by adding a deep learning model to a general camera, but it has been resolved through Intel’s Realsense D435 camera module for fast computation [46]. This method aims to confirm the validity of the data according to the proposal and the use of the joint coordinate correction and computational algorithm required for overall content production using the deep learning process. As for the content, the content related to the operation that the demo program may perform and the design content are displayed, and a test performance method is described.

The execution of the program calculates the angle data of the joint using the following method.

**Figure 4:**
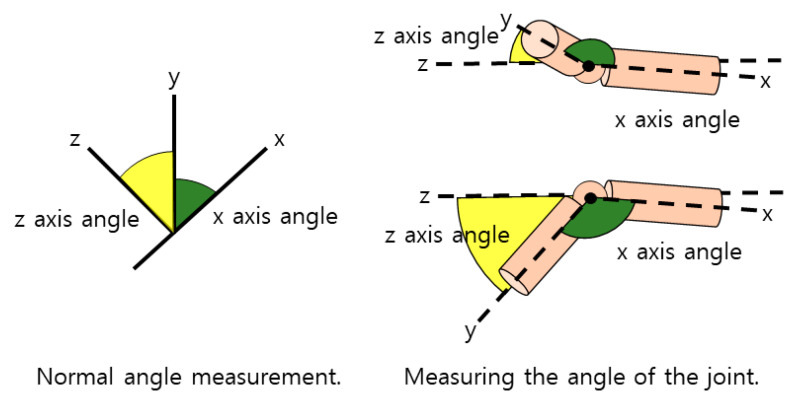
Image of how to measure joint angles and general methods.

Figure 4 is an image that arbitrarily shows the measurement of a general angle and the measurement at the joint with respect to measuring the angle of the joint. Unlike the general method of calculating the angle and direction based on the three coordinates, the proposed angle on the program means measuring the angle in multiple directions. The reason for using this operation is to accurately measure the rotation angle of the joint that may vary according to the camera angle required for photographing. Assuming that there is a virtual plane according to the slope in the corresponding direction, the calculation of the angle moving from the plane is performed, and the angle is measured using data corresponding to the three axes [47]. In addition, in the case of patients during joint motion, unlike normal people, the joint rarely rotates beyond normal motion. Therefore, in addition to activities such as flipping the body, abnormal motion detection exceeding 90 degrees or 180 degrees is excluded, except for joints that are unfolded when performing general movements.

**Figure 5:**
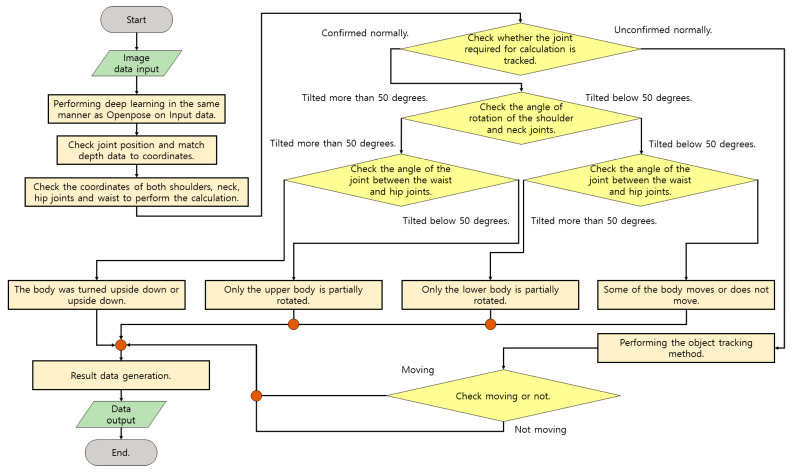
Flow chart of the system to prevent bedsores.

Figure 5 is a flowchart illustrating the execution of bedsore prevention and movement confirmation to be performed in the system. This content is mostly performed for patients who lie down to relax and recover. The main functions provided are to check the inactive time to prevent patient bedsores and to check whether the body has been moved. First, real-time motion is photographed with a camera. The captured data measures coordinate data of the joint through a deep learning model. In general, it is necessary to check whether the body has moved, turned completely upside down, or stayed still by checking the patient’s condition through object recognition or classification methods. However, human joints can only rotate the lower body or lie on the side of the upper body, and the amount of movement is measured differently depending on the degree of discomfort of the patient.

**Figure 6:**
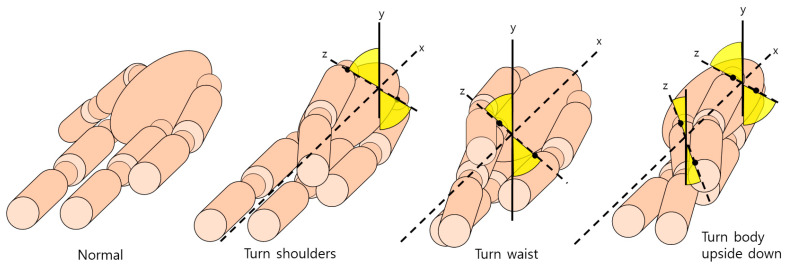
An image indicating a method of detecting the rotation direction of the hip joint based on the spine.

Figure 6 shows the operation of identifying the coordinates of the corresponding joints set as a reference and measuring the joints of the aforementioned method through one other joint connected to the joints. The narrower the part marked with the angle in yellow, the greater the movement, and the movement is classified according to the angle of the joint. When checking the angle of the two joints of the pelvis around the central axis of the bone between the neck and pelvis, body movement is judged to be moderate when it progresses more than 45 degrees, and the reason for this is that the body cannot hold out after it has already risen to some extent. Based on the central axis, checking whether there is a degree of movement when lying down is made according to the rotation of the pelvis. In order to prevent erroneous detection when proper data input is not received during the operation, it is checked whether the operation has been performed well using the object detection method.

**Figure 7:**
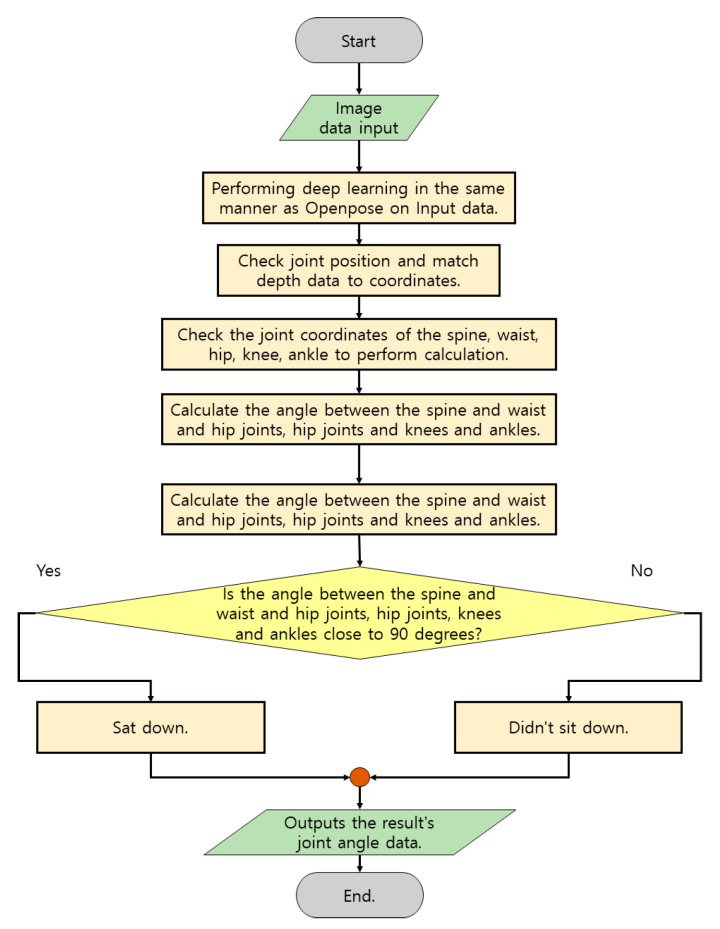
Flow chart image performed to check the joint angle of the leg.

Figure 7 is a flowchart of the performance of checking the range of motion of the leg joint to be performed by the demo program. This content is mainly performed for patients who can move standing and need to be careful about their leg movements. As a function provided, information about movements such as the angle and direction of progress of the patient’s leg can be checked so that the patient’s leg condition and degree of recovery can be ascertained. In addition, if patients who are living lying down also need to check the recovery of their legs, it is possible to perform measurements to check the recovery of both legs by raising and lowering their legs. As with the previous method, real-time camera photographing is performed and measurement is performed by photographing from the front or diagonal side. The angle of the joint calculates the angle between the waist and hip joint and the knee, and the angle between the hip joint and the knee and the ankle. The initial leg data is between 175 and 180 degrees due to the degree of straight joints, knees, or hip joints. However, it gradually approaches 90 degrees in the sitting position, and on the contrary, even when calculated, the data rises from 0 degrees to 90 degrees. In the case of hip joints, the same angle data is shown.

The following is the part where an algorithm was added to the problem in designing the demo program. Since limited color information is used in the patient’s living space inside the hospital, there is not much data information that can be distinguished during the calculation of deep learning. Therefore, there is no problem in photographing data, but there is a phenomenon in which recognition data is bounced to a place unrelated to some image frames.

**Figure 8:**
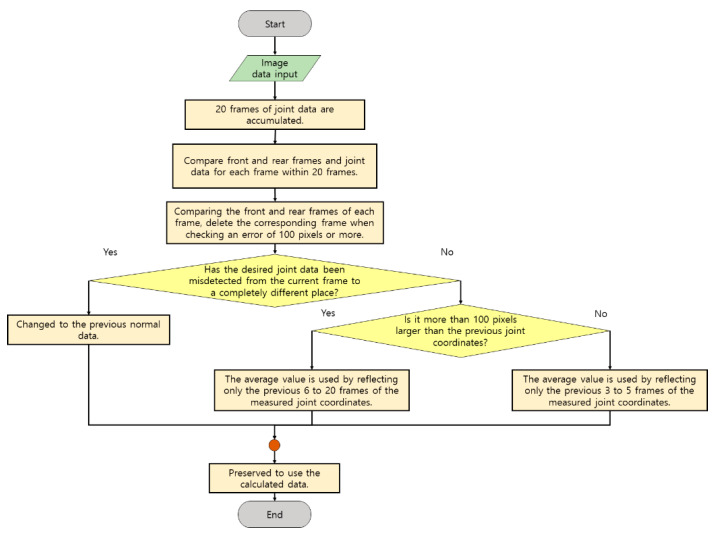
A flowchart of a method of stabilizing data when detecting an object.

Figure 8 is a method used to level misdetection data in an image frame. The method stores the previous data frame separately for approximately 20 frames and averages the estimated joint data within a certain 20 frames by comparing it with the current joint data. In addition, the location of the adjacent joint is checked, and data at completely different coordinate points are not used for computation, or previous data is used. If the data of the previous frame is close to the data of the current frame, it is judged that there is a problem with operating more than 100 pixels in one frame as the data of the error, and although it is reflected, it is determined how much the frames accumulated before that are reflected. After that, it is reflected in the calculation based on the refined data.

Examples 1 and 2 of the following equations are as described above:

(1) $$y=\{\begin{array}{cc} 6\sim 20\;(\mathrm{When it is confirmed that there is no false detection data}.)\\ \; & 3\sim 5\;(\mathrm{When some coordinate data is in the wrong position}.)\\ \; & 0\;(\mathrm{When most of the coordinate data is incorrectly detected or}\\ \; & \mathrm{there is no accumulated data required for calculation}.) \end{array}$$

(2) $$\frac{1}{y}\overset{y}{\underset{x=1}{\sum }}frame\;x\;joint\;data$$

The contents of formulas (1) and (2) represent the contents before the equation, and in formula (2), ‘*frame x point data*’ means the data coordinates of each joint for each frame. For coordinate correction, formula (2) is applied to each joint and used.

Table 3 summarizes the test method to make it easier to understand. The first method is to perform an action to prevent bedsores. The lying person performs a right and left turn around and checks how much the measured data rotates through the hip and waist joints. The second way is to perform the sitting motion of the standing person and check the data on how much the joints of the entire leg worked from the measured frame to the hip and knee, knee and ankle.

## 4. Results

The following restrictions were followed regarding the performance of the test:

- While conducting the test, the installation location of the cameras and the environment in hospitals were limited due to the Medical and Personal Information Protection Act in Korea;
- Consent from the hospital or medical staff was obtained, and the face was mosaiced in the case of the patient;
- In the case of the shooting angle, it was limited to the bed. Data obtained after the test were also provided to hospitals for patient rehabilitation;
- The test to perform the proposed motion may not be possible, so it was performed with the consent of two people and two ordinary people who recovered to some extent after surgery.

The overall performance method is a test performed to identify a data collection method suitable for the Korean medical system and to clearly grasp the data desired by medical staff. In addition, tests were conducted to confirm the results of whether significant angular data were identified through the system and to what extent the patient’s condition could be identified.

**Figure 9:**
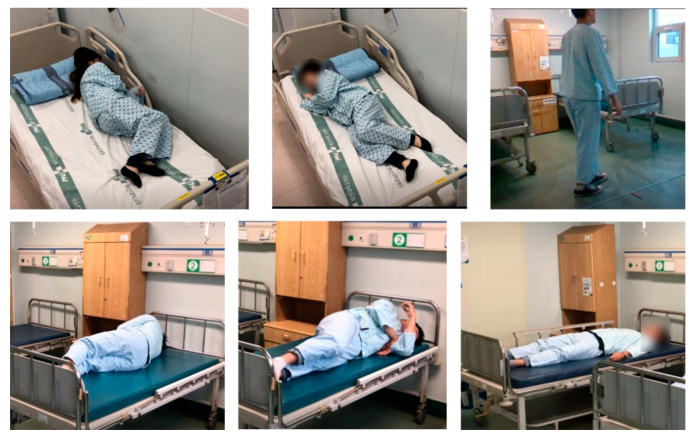
The image of the hospital room and patient in various positions from different directions.

Figure 9 is an image of the results taken in various directions using a camera to check the measurement range. While taking the image in the hospital room, the environment was checked to see if the proposed process could work well. There is no problem with filming itself and people are easily detected, but when there is a joint temporarily covered by the body, Z-axis data to generate coordinates required for angle measurement is not detected. For patients who usually lie down, the camera should be installed to look down from the ceiling, but in Korea, it has been confirmed that it would be ideal to have the camera look diagonally downward in the form of a high-positioned IV due to privacy security problems. As a method, one person’s measurement is performed, and the use of the program in an environment where a large number of people are divided is a study that can be conducted later. Hospitals require a method of installing hardware that can normally perform programs without disturbing patients and medical staff [48,49,50]. Therefore, before performing the program, the angle and location in a general hospital environment where image data can be photographed were checked, and the measurable range was confirmed through a simple operation. These movements were measured separately between the normal person and the patient, and the motion data of the normal person and the patient were compared and confirmed. In order to analyze the data, a separate calculation program was produced to convert the coordinates of the demo program into a real angle used in the hospital, and the converted angle data could be stored if necessary.

**Figure 10:**
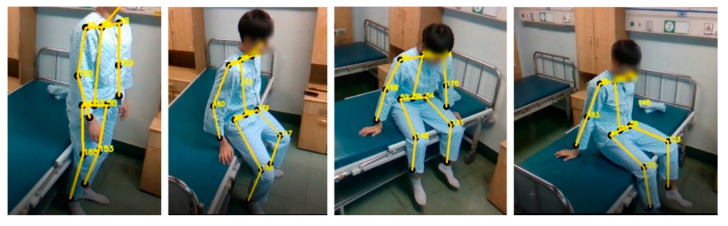
Measurement image of the sitting motion.

Figure 10 is an image of the sitting motion measured using a program to check the motion state of the leg. Joints corresponding to the ankles from the waist to the knees and knees were identified as the target of exercise when sitting, and joint angle data were checked using related coordinate data. The movement was not performed until the end, and for safety reasons, it was performed by sitting on the bed if the patient was likely to fall.

**Figure 11:**
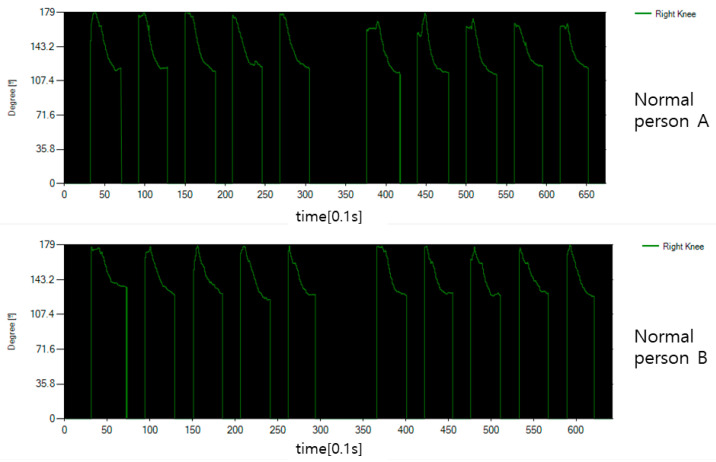
The knee angle data of a normal person’s sitting motion.

Figure 11 is a graph showing the angle expressed around the joint corresponding to the knee for the sitting motion of a normal person. In normal condition, the legs are stretched close to 180 degrees, and when the knees are bent to sit, they are significantly reduced to approximately 120 degrees.

**Figure 12:**
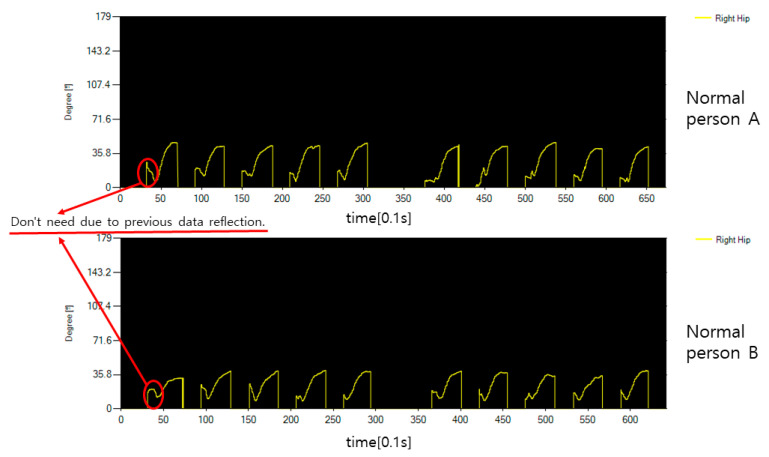
The hip bone angle data of a normal person’s sitting motion.

Figure 12 shows the angle expressed around the joint corresponding to the hip bone. Since moving the thigh is a forward movement, it starts at close to 0 degrees and the angle gradually rises. In the data graph, incorrectly input data for each operation is data reflected in the waiting time between operations, and real angle data rises after going down. In Figure 11 and Figure 12, the normal person shows a clean form without the data shaking in motion.

**Figure 13:**
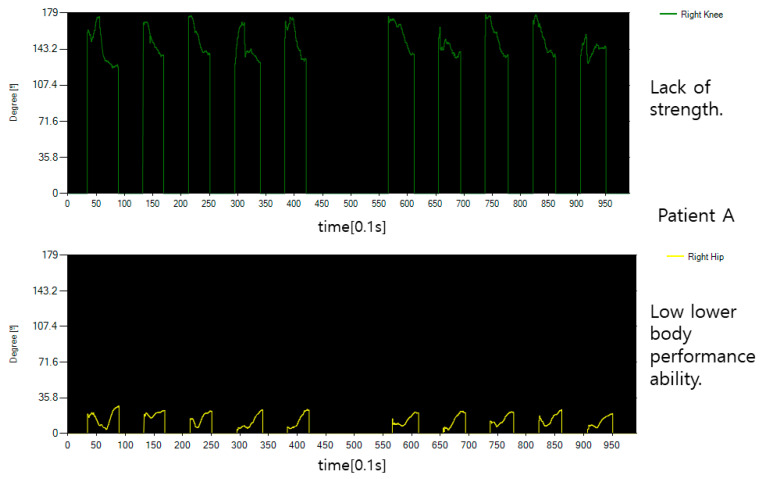
The angle data of patient A sitting motion.

Figure 13 shows the behavior of patients who need lower body rehabilitation. First, what is clearly confirmed is that the overall data is not clean and has a lot of tremors, which indicates a lack of force. In addition, since I lies in bed because I cannot sit properly, the angle on the hip bone side does not change much. 

**Figure 14:**
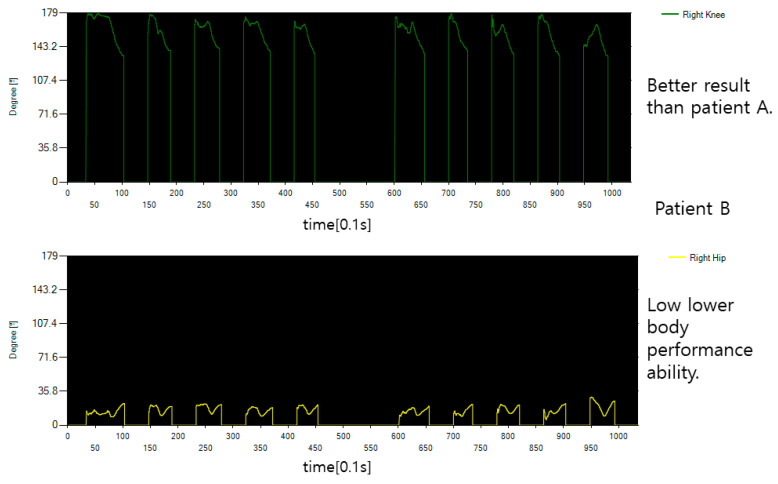
The angle data of patient B’s sitting motion.

Figure 14 shows the behavior of other patients who need rehabilitation in the lower body as shown in Figure 13. The fold of the knee is a little better than patient A, but the hip bone or waist joint is not used properly and does not bend significantly. The data from the normal person shown are markedly different.

**Figure 15:**
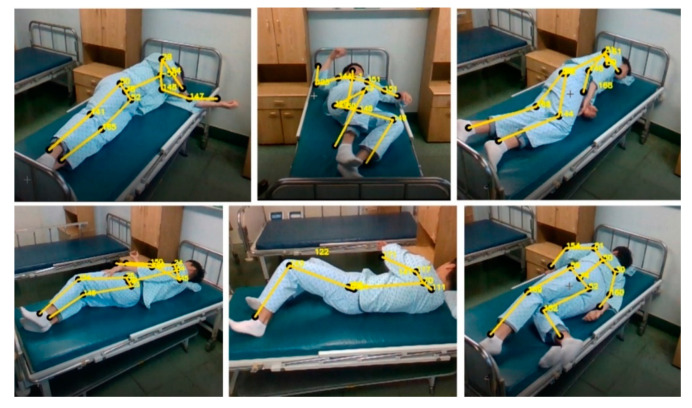
Measurement image of the motion of rotating the body while lying down.

Figure 15 shows the measurement of lying down and turning the body from various angles with a system to prevent bedsores. The motion is not measured at the angle shown in the actual figure but is confirmed through the rotation of the hip joint centered on the spine. The direction of measurement was measured at different locations for each measurement or for each person.

**Figure 16:**
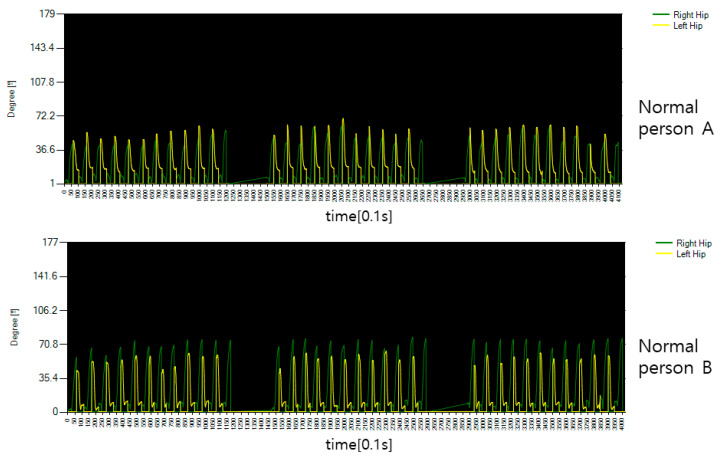
The hip joint angle data of the motion that rotates the body of an average healthy person.

Figure 16 shows the rotation angle between both hips and spine for the movement of flipping the body of the general public. Looking at the graph, you can see that both hips rotate in different directions for each operation time, which means that the rotation directions of both hips are different. In performing this movement, if the body is moved above 45 degrees, it is determined that it has been moved to some extent, but in the case of normal people, it is possible to flip the body 45 degrees or more, indicating that the overall performance was good.

**Figure 17:**
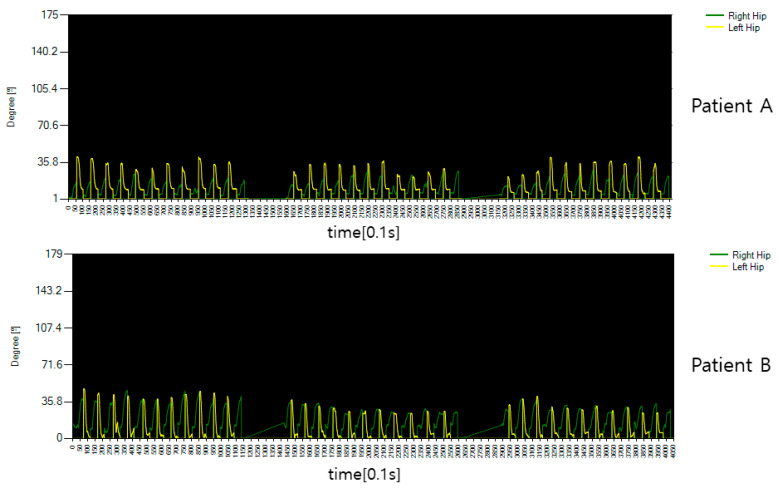
The hip joint angle data of the motion that rotates the body of a patient.

Figure 17 shows the rotation angle between both hips and spine for the motion of rotating the patient’s body. It can be seen that it is significantly different from the general public in Figure 16, but it was confirmed that it was passive because it was accompanied by pain in performing the movement and that it was too much to rotate after a certain movement. This is a measure of the angle of movement of the body, and the result is that there is a lack of power to turn the body to an angular section of 45 degrees or more determined by flipping.

This experiment was conducted by producing a demonstration program to check whether a movement, practically performed through a sitting motion, in a patient who must spend time lying down without moving, can prevent bedsores and easily check their leg health. The program detected joints from images using a learning model through deep learning and establish whether it could be used as a stepping stone to help hospitals monitor recovery status and specific motion performance.

Table 4 shows the test reference angle for performing the test and the average angle for each operation. In the case of the average angle, angles within the range of motion effective in the joint angle graph after the test was performed were used. The conventional measurement method was measured using a protractor. As a result of the test, it was confirmed that the joint angle criteria that the patient could perform were designated, and the angle was estimated after performing certain movements.

**Figure 18:**
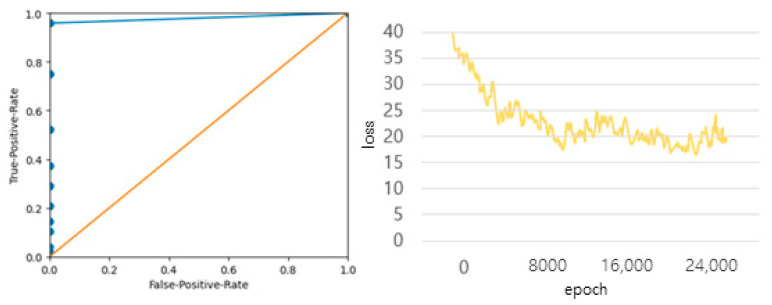
ROC curves and joint data learning loss curves for 50 joint angle data based on error values exceeding 5 degrees.

Figure 18 shows the ROC curve that compares the joint angle data used during the test through the actual towing machine to check the error and shows the ‘negative’ when the error occurs more than 5 degrees, and the loss curve when learning joint data deep learning. Some joint data were misdetected by partially reflecting the data before movement, but more than 95% of the important medical data were available except for data with large errors in motion, and it was confirmed that the difference in joint angle was within 5 degrees.

Table 5 compares the joint angle measurement method and the proposed method. For actual measurement, medical staff directly check the performance of motion through a protractor and draw and measure it using a program similar to that of a picture plate, so it can be seen that the proposed method is performed very quickly. In addition, it can be used very flexibly because it can be performed directly without a medical staff member next to it. The movements are related to rehabilitation and consist of movements that can be performed alone without difficulty, allowing the daily monitoring of movable joint data. Medical staff can measure data more easily and continuously than before, which is processed in real-time. Rehabilitation must be carried out steadily, which means continuous treatment for the patient. Although it cannot directly replace major medical personnel, it has been confirmed that there is no problem in temporary assistance or content production for patients.

## 5. Conclusions

In this paper, a method of designing a related content demo diagnosis system for the rapid recovery of patients after surgery using deep learning image processing technology was studied. As for the related demo content, it was produced to check the degree of rotation of the body to prevent bedsores or to see how much the condition of the patient who cannot move his or her legs properly has improved. In fact, the angles for measuring motion were adjusted by referring to related medical books or consulting rehabilitation medical personnel so that they could be identified in the form of general-purpose data used by Korean doctors. Doctors also focused on accuracy in performing deep learning, the results were confirmed by explaining them as diagnostic or rehabilitation assistance in the performance method, and the development for the introduction of related systems was positively evaluated. However, related motion content was additionally required, and in order to solve this problem, accurate posture advice from an expert was required. There are still aspects that cannot be replaced by technology and areas where doctors’ standards of judgment are essential. However, there are certainly areas that need to be improved for assistance. Conventionally, doctors have high reliability due to using a method of measuring with a direct protractor to check the angle of the joint motion of patients, however, have not solved the problem of taking too long for each movement.

As a result of performing the test, the accuracy of confirming the joint direction and motion of each joint was quite accurate. Explaining to the patient the relevant posture, and how to check whether it is performed accurately, seems to be a satisfactory temporary replacement, as is leaving it to the system and checking other patients. In addition, it is possible to check the state of recovery, although the performance intensity varies depending on the patient’s condition, by storing the existing data and comparing it in the next performance. It was performed faster than the other methods, such as being able to check the actual angle, checking the behavior of the problematic patient with the eyes, or using a protractor. In order to further increase related content in the future, it is devising a way to improve the accuracy of the action by securing data related to the classification of movements that can be performed without the help of medical staff, converting deep learning model layers and correcting coordinates through the introduction of two or more cameras.

## Figures and Tables

**Table 1:** A diagnostic solution using deep learning used in Korea.

	Usage Data	Diagnosis Category	Utilization
VUNO Solutions	Medical image	Some lesions that can be expressed in X-ray.	Available
IBM Watson	Symptom (Text)	Cancer	Unavailable

**Table 2:** The difference between human pose estimation methods.

	Motion Capture Sensor	Depth Camera	Normal Camera
Performance	High	Middle	Low
Convenience	Bad	Good	Good
3D axis measurement	O (No need for additional functions.)	O (No need for additional functions.)	X (Additional function required)
Required Time	Long	Short	Short
Technical difficulty	High	High	Very high
Cost	High	Middle	Low

**Table 3:** How to perform the test.

	Test Method	Goals	Movement	Measuring Joints	Exceptions
1	Check the radius of waist rotation.	Prevention of bedsores.	Turn body in both directions while lying down.	Both hip joints and waist-centered joints.	Angle and location of camera shooting in accordance with the Korean Medical and Personal Information Protection Act. Patients who cannot perform any motion at all.
2	Check the radius of rotation of legs.	Check the leg movement.	Slowly sit on the bed.	The knee angle from the hip joint to the ankle angle.	

**Table 4:** Test criteria and average angle of each test motion.

	Normal Angle (Criterion)	Normal Person A (Average)	Normal Person B (Average)	Patient A (Average)	Patient B (Average)
Sitting down slowly motion	Knee: 130°	Knee: 115°	Knee: 121°	Knee: 143°	Knee: 151°
Lying down and turning sideways motion	Waist: 45°	Waist: 68°	Waist: 66°	Waist: 29°	Waist: 31°

**Table 5:** The difference from the existing method.

	Proposal	Existing
Form	Program (Automatic)	Protractor (Hand measurement)
Data collection	Automatic	Handwritten
Measurement	Real-time or as soon as move	While maintaining the movement
number of people required	Can be done alone	Need medical staff
Time required	Immediately or operating time	About 30 min
